# Mechanical Properties of Basalt–Polypropylene Hybrid Fiber-Reinforced Red Mud–Coal Metakaolin Geopolymer

**DOI:** 10.3390/ma19081578

**Published:** 2026-04-14

**Authors:** Jiuyu Zhao, Guangzhong Yu, Luorui Hu, Yinghao Dong, Haoran Liu, Chao Guo, Yongbao Wang

**Affiliations:** 1 College of Civil Engineering, Taiyuan University of Technology, Taiyuan 030024, China; 2023002519@link.tyut.edu.cn (J.Z.); 2023002565@link.tyut.edu.cn (G.Y.); 2023002548@link.tyut.edu.cn (L.H.); dyh2382617785@gmail.com (Y.D.);; 2 School of Infrastructure Engineering, Dalian University of Technology, Dalian 116024, China; liuhaoran@mail.dlut.edu.cn

**Keywords:** geopolymers, red mud, fibers, strength, response surface

## Abstract

Red mud-based composites show great potential in industrial solid waste utilization in response to the growing demand for low-carbon building materials. However, red mud–coal metakaolin geopolymers (RCGs) exhibit high brittleness and poor crack resistance, which limit their application in practical engineering. In order to improve the strength and toughness of RCGs, this study proposes a hybrid reinforcement strategy combining basalt fiber (BF) and polypropylene fiber (PPF). Effects of fiber length and fiber content on the mechanical properties of RCG were systematically investigated by orthogonal experimental design and response surface methodology (RSM). The microstructural characteristics were also analyzed using SEM, EDS, and XRD. Results show that fiber incorporation effectively enhances the mechanical properties and toughness of RCG, and BF length is the key factor influencing the strength of RCG. The optimal fiber ratio (BF: 11 mm, 0.23%; PPF: 6 mm, 0.20%) increases 9.52% of 28-day compressive strengths and 18.93% of 28-day flexural strengths. Microstructural analysis shows fibers bridging, interfacial stress transfer, and pull-out, which inhibit crack propagation. However, excessive fiber content may reduce matrix continuity. This manuscript provides a theoretical basis for optimizing red mud-based geopolymer composites and promotes the resource utilization of industrial solid waste.

## 1. Introduction

Driven by global carbon neutrality and carbon peaking goals, as well as the demand for solid waste resource utilization, geopolymers have attracted significant attention due to their low-carbon, environmentally friendly nature and excellent performance [1,2,3,4]. Red mud (RM) is a large amount of alkaline waste generated in the alumina industry [5], rich in silicon, oxygen, aluminum, and other components [6], making it a potential precursor for geopolymers [7], and coal metakaolin (CMK) can enhance reaction activity [8]. The synergistic preparation of geopolymers by the two can realize the resource utilization of solid waste, reducing costs and carbon footprint, making them promising green material [9].

Red mud–coal metakaolin geopolymer (RCG) belongs to the class of inorganic aluminosilicate cementitious materials. Its strength is usually not very high and the resistance to cracking is also limited, which makes its use in load-bearing engineering structures difficult [10]. Some studies have suggested that modifying the raw material proportions and the modulus of the alkaline activator can increase the compressive strength to a certain extent [11]. Guo et al. [12] indicated that the compressive strength of RCG reached its peak when the alkali activator modulus was 1.4, but its flexural strength and crack resistance still needed improvement [13,14]. Fiber reinforcement is recognized as an effective approach to improve brittleness and enhance overall performance. It significantly increases material ductility by bridging cracks and dissipating energy. Previous studies have mainly considered the use of a single fiber for reinforcement. Gao [13] observed that adding polypropylene fibers (6 mm in length, 0.5% by volume) improved the flexural strength, whereas the compressive strength was almost unchanged. Wang et al. [15] examined different fibers, including glass fiber, basalt fiber, polypropylene fiber, and polyvinyl alcohol fiber. Their results showed that a single fiber type generally cannot achieve improvements in both strength and toughness at the same time. Among them, different types of fibers play different roles: rigid fibers mainly play a crack-bridging role, while flexible fibers can dissipate energy during crack propagation. The synergistic effect of hybrid fibers can more effectively inhibit the generation and expansion of microcracks, thereby significantly improving the mechanical properties, crack resistance, and toughness of geopolymer composites [16]. Previous studies mostly examined conventional cement-based materials [17] and red mud–cement composites [18], focusing on the performance improvement methods of concrete with high red mud content (>20%), solving the problem of concrete strength reduction caused by high red mud content through methods such as adding fibers, high-temperature treatment, and steel slag addition. However, most of them only mention the effect of single fibers on the strength, toughness, and crack resistance of red mud concrete, and there are few studies on mixed fibers under high red mud content. Fiber hybridization provides synergistic mechanical enhancement that overcomes the limitations of single fibers, thereby improving strength, crack resistance, and toughness. By combining high-performance and low-cost fibers, it achieves a balance between performance and cost while maintaining effective reinforcement. In addition, hybridization enhances fiber–matrix interfacial bonding, promotes efficient stress transfer, and reduces interfacial debonding.

At present, there remain a series of research gaps in the field of hybrid fiber reinforcement. For RCG, the effects of fiber length and volume fraction on mechanical properties, the relative importance of these factors, and the underlying microstructural reinforcement mechanisms still remain insufficiently understood. Excessive fiber dosage may also lead to fiber agglomeration [19], increasing internal voids in the matrix [20], and reduce the strength. Besides, there is also a lack of systematic optimization methods for the design of hybrid fiber composites. Existing studies mostly rely on empirical mix proportions, and a quantitative relationship model linking “fiber type–hybrid ratio–matrix performance” has not been established. This fails to provide scientific guidance for the formulation of fiber hybrid schemes in engineering practice, making it difficult to fully exploit the advantages of fiber hybridization.

To determine the appropriate fiber combination, previous studies on different fiber types were reviewed to guide the selection. The inclusion of steel fibers has been shown to significantly enhance the flexural strength, toughness, and post-cracking load-bearing capacity of high-performance geopolymers. Through a crack-bridging mechanism, steel fibers effectively inhibit crack propagation and augment the material’s energy absorption capacity. Although steel fibers can significantly boost the strength and toughness of geopolymers, they suffer from high density, susceptibility to corrosion, and poor dispersibility, this often leads to issues such as agglomeration or the formation of “fiber balls”, resulting in reduced workability and an increase in internal defects. Furthermore, the ability of a single type of steel fiber to synergistically control multi-scale cracks is limited, making it difficult to simultaneously achieve a comprehensive enhancement of both strength and toughness [21]. Some studies have also explored the incorporation of natural fibers, such as coir and sisal, which have been shown to effectively mitigate plastic shrinkage cracking and improve the residual load-bearing capacity post-fracture. However, the efficacy of this approach is contingent upon the dispersibility of fibers and the quality of their interfacial bonding, and the improvement effect is not stable in actual use [22].

To achieve a complementary effect, this study employs basalt fibers and polypropylene fibers as reinforcement materials and conducts a comparative investigation based on their significant performance differences. Basalt fibers demonstrate excellent mechanical properties, high stiffness, and superior chemical stability, while polypropylene fibers are characterized by high toughness, low density, and favorable workability during mixing; the combination of them provides synergistic reinforcement by integrating high stiffness with ductility, thereby improving strength and crack resistance. This study adopts RM and CMK as composite precursors and introduces a hybrid reinforcement approach using basalt fiber and polypropylene fiber. The influence of fiber length and volume fraction on the strength of RCG at different curing ages was evaluated. The Response Surface Methodology (RSM) was used for strength prediction. Meanwhile, the synergistic strengthening mechanism of the fibers was further revealed through microstructural methods such as SEM, XRD, and EDS, providing a scientific basis for material optimization design.

The academic novelty of this study lies in the simultaneous incorporation of basalt fibers and polypropylene fibers into the same red mud–coal metakaolin geopolymer system, enabling a systematic comparative investigation of their composite performance. Furthermore, through quantitative analyses the percentage increase in geopolymer strength attributed to fiber volume fraction and length was determined. By optimizing the dosage and synergistic interaction of the two types of fibers, a balanced enhancement of strength and toughness can be achieved. Furthermore, through comprehensive testing of compressive and flexural properties, combined with statistical analysis and microstructural characterization, this study also provides an understanding of the fiber–matrix interfacial mechanisms, offering a new perspective on the synergistic reinforcement of blended fibers in red mud-based geopolymer systems.

## 2. Materials and Methods

### 2.1. Raw Materials

**Figure 1:**
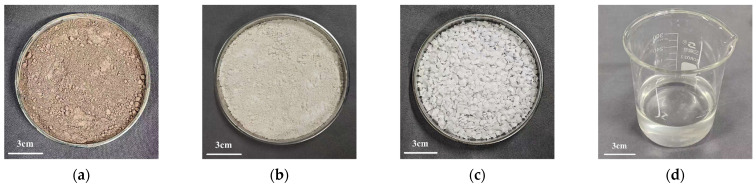
Photographs of raw materials: (**a**) RM; (**b**) CMK; (**c**) NaOH solid; (**d**) sodium silicate solution.

**Figure 2:**
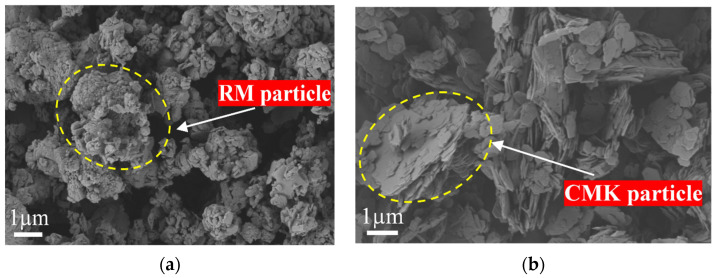
SEM images: (**a**) RM; (**b**) CMK.

RM, also referred to as bauxite residue, is a highly alkaline industrial by-product generated during alumina extraction through the Bayer process. In this study, the RM was collected as a solid residue generated during the clarification stage following bauxite digestion in the Bayer process at an aluminum plant in Hejin, Shanxi Province, China. Figure 1a and Figure 2a show the macroscopic and SEM images of RM. The raw RM blocks were first crushed and ground to 200 mesh. CMK was produced by Xinzhou Jinyu Industry and Trade Co., Ltd., Xinzhou, Shanxi Province, China. It has a white-yellow powder appearance and a particle size of 2 μm. Unlike standard metakaolin, CMK is derived from coal gangue or coal-bearing kaolinite and may contain more impurities. However, after calcination, it transforms into a highly reactive amorphous aluminosilicate phase and, due to its lower cost relative to conventional metakaolin, can be used as an alternative precursor in geopolymer systems; macroscopic and SEM images are shown in Figure 1b and Figure 2b. The main chemical components of RM and CMK are shown in Table 1, indicating high pozzolanic activity. The flake-shaped NaOH was supplied by Kaitong Chemical Reagents Co., Ltd., Tianjin Municipality, China, as shown in Figure 1c, with a purity of 95%. Figure 1d shows the appearance of the sodium silicate solution, which was produced by a building materials factory in Shanxi Province, with mass fractions of 26.2% SiO_2_ and 8.3% Na_2_O and an initial modulus of 3.20.

**Figure 3:**
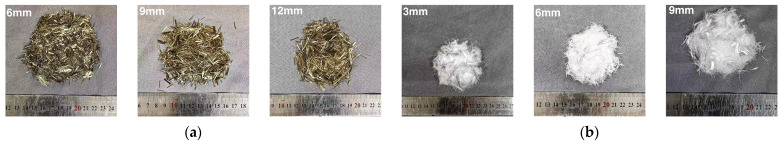
Appearance of fibers: (**a**) Basalt fibers of 6 mm, 9 mm, and 12 mm; (**b**) Polypropylene fibers of 3 mm, 6 mm, and 9 mm.

The alkaline activator is formulated from NaOH and sodium silicate solution, with a modulus of 1.4. After blending, the blend was rested for 12 h to ensure consistent alkaline activation [23]. Experimental fibers were sourced from basalt chopped strand fiber (BF) produced by Haining Anjie Composite Materials Co., Ltd., Haining, Zhejiang Province, China, and bundled polypropylene monofilament fiber (PPF) produced by Jinan Shunjie Engineering Materials Co., Ltd., Jinan, Shandong Province, China. The appearance and physical-mechanical properties of both fibers are presented in Figure 3 and Table 2. The data in the table are provided by the manufacturer. BF with lengths of 6, 9, and 12 mm and PPF with lengths of 3, 6, and 9 mm were prepared for use. Tap water from Shanxi Province was used as the mixing water.

### 2.2. Experimental Protocol Design

This study used an orthogonal experimental design, maintaining a constant geopolymer mix ratio across all test groups. According to Liu et al., the elemental molar ratios were set at Si/Al = 1.2 and Na/Al = 1.0 [24], with a liquid-to-solid ratio of 0.55, an alkaline activator modulus of 1.4, and a coal metakaolin-to-red mud mass ratio of 3:7 for preparing RCG specimens [25]. The BF length (6 mm, 9 mm, 12 mm), PPF length (3 mm, 6 mm, 9 mm), BF volume fraction (0.1%, 0.2%, 0.3%), and PPF volume content (0.1%, 0.2%, 0.3%) were designated as factors A, B, C, and D, respectively. An L9(3^4^) orthogonal design was employed to establish 10 experimental groups for curing ages of 7 and 28 days, including a control group. The factor levels and specific experimental details are presented in Table 3.

### 2.3. Specimen Preparation and Testing

**Figure 4:**
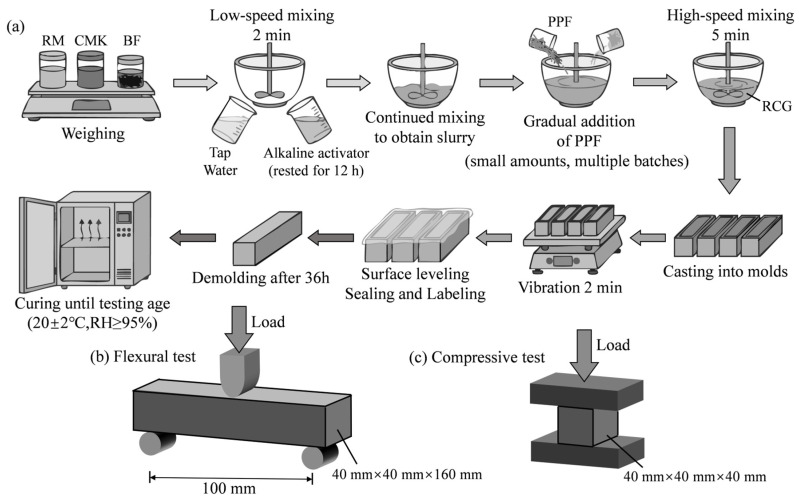
Preparation procedure and mechanical test setup: (**a**) preparation procedure; (**b**) flexural text; (**c**) compressive text.

The rated masses of RM, CMK, and BF (pre-dispersed) were added to a mortar mixer and mixed at low speed for 2 min to ensure uniform fiber dispersion. After thorough mixing, tap water and the alkali activator were slowly added while stirring continued until a slurry with good flowability was obtained. Pre-dispersed PPF was then introduced in small batches. Finally, the mixture was stirred at high speed for 5 min to form a fiber-reinforced geopolymer slurry. The slurry was poured into molds with dimensions of 40 mm × 40 mm × 160 mm. The molds were then placed on a vibrating table and vibrated for 2 min to remove entrapped air bubbles. The surface was leveled using a scraper, and the molds were then covered with plastic wrap. After 36 h of curing, the specimens were demolded, then the specimens were wrapped in plastic wrap and placed in a curing chamber maintained at 20 ± 2 °C and RH ≥ 95% until the designated testing ages of 7 and 28 days. The preparation process is shown in Figure 4a.

A total of 20 groups of specimens were prepared, including 10 groups for each curing age. For each group, three specimens were prepared and tested for mechanical properties (compressive and flexural strength), and the reported results represent the average of these three measurements. To ensure the reliability and reproducibility of the results, the entire experimental program was repeated eight times. According to GB/T 17671-2021, China [26], a 600 kN hydraulic servo universal testing machine was used to test the flexural strength and compressive strength of the specimens (Figure 4b,c), with loading rates of 2400 N/s and 50 N/s, respectively.

The central portion of the fractured specimens was selected for SEM analysis. The samples were first treated with anhydrous ethanol to remove moisture and stop further hydration, followed by drying. Small pieces were then cut from the fracture surface, mounted on conductive adhesive, and sputter-coated with a thin layer of gold (approximately 5–10 nm) to ensure sufficient surface conductivity prior to SEM observation with a Gemini SEM 300 scanning electron microscope, Zeiss, Oberkochen, Germany. In addition, energy dispersive spectroscopy (EDS) analysis was performed on the fiber–matrix interface region. For XRD analysis, the central portion of the specimen was taken as the sample, crushed and ground into powder, and then sieved to obtain the XRD sample. Phase composition analysis was performed using a Panalytical Empyrean X-ray diffractometer, PANalytical, Almelo, The Netherlands.

### 2.4. Statistical Analysis

To evaluate the significance of the influencing factors on the mechanical properties of RCG, analysis of variance (ANOVA) was conducted on the experimental results. The significance level was set at α = 0.05. The F-value was used to determine the statistical significance of each factor.

## 3. Results

### 3.1. Strength Results

#### 3.1.1. Compressive Strength

**Figure 5:**
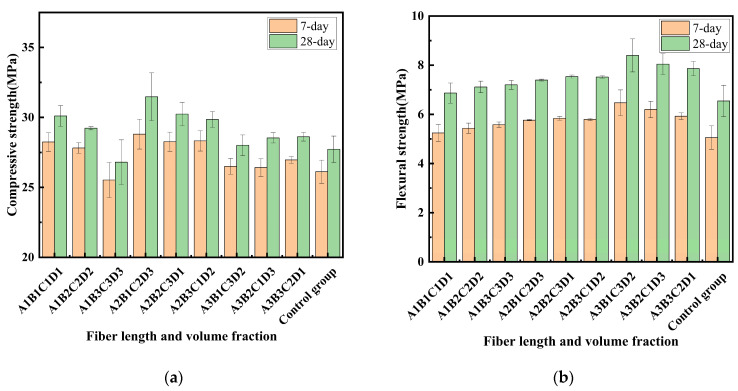
Mechanical strength of specimens: (**a**) compressive strength; (**b**) flexural strength.

The compressive strength results of the RCGs are presented in Figure 5a. Compared with the control group, most fiber-reinforced specimens showed improvement in compressive strength, although the magnitude of improvement varied among different groups. This indicates that fiber incorporation can be beneficial to compressive performance, but the reinforcing efficiency strongly depends on the selected fiber parameters.

The compressive strengths of the control group at 7 and 28 days were 26.1 MPa and 27.7 MPa, respectively, corresponding to a 6.1% increase with curing age. For the other experimental groups, the strength increases from 7 to 28 days ranged from 5.6% to 8.9%, with an average value close to that of the control group. This suggests that fiber incorporation has little influence on the age-dependent development trend of compressive strength. At low to moderate fiber contents, the improvement in compressive strength can be attributed to the combined effects of matrix densification and fiber reinforcement. On the one hand, well-dispersed fibers help restrain crack development and transfer stress across microcracks. On the other hand, when the fiber content remains within an appropriate range, the continuity and compactness of the geopolymer matrix can still be maintained, which is favorable for load-bearing capacity under compression.

However, when the fiber dosage becomes excessive, the compressive strength decreases. Compared with the control group, the A_1_B_3_C_3_D_3_ group exhibited the lowest mechanical performance, with the 7-day compressive strength decreasing by 2.25% and the 28-day compressive strength decreasing by 3.34%. This reduction is mainly attributed to the loss of matrix integrity caused by excessive fiber incorporation. Specifically, fiber agglomeration and poor dispersion may introduce internal voids and local defects, reduce matrix compactness, and hinder effective stress transfer [27,28]. Under compressive loading, these adverse effects can outweigh the crack-bridging contribution of the fibers, resulting in reduced compressive strength.

Among all groups, A_2_B_1_C_2_D_3_ showed the highest compressive strength, and its 28-day compressive strength was 13.5% higher than that of the control group. In addition, the 28-day compressive strength of A_2_B_2_C_3_D_1_ was 9.1% higher than that of the control group. These results indicate that the hybrid incorporation of BF and PPF can effectively improve the compressive performance of RCG when the fiber parameters are properly controlled. From a mechanistic point of view, the strengthening effect does not arise solely from fiber bridging. Instead, it is governed by the balance between matrix densification and fiber reinforcement efficiency. BF, owing to its relatively high stiffness, mainly contributes to macroscopic load bearing and crack bridging, whereas PPF, with better deformability, helps restrain the initiation and propagation of microcracks. When the fiber content and length are appropriate, these two fibers can play complementary roles without significantly disturbing matrix continuity, thereby leading to higher compressive strength.

#### 3.1.2. Flexural Strength

The flexural strength results of all test groups are presented in Figure 5b. A similar evolution trend is observed at both curing ages, with the 28-day flexural strength consistently higher than the corresponding 7-day value. However, the increase with curing age is relatively limited, indicating that the flexural performance of RCG is mainly characterized by early strength development. Compared with the control group, all fiber-reinforced specimens exhibited improved flexural strength [19], indicating that fiber incorporation is generally beneficial to the bending resistance of RCG. Among all groups, A_3_B_1_C_3_D_2_ showed the most significant improvement, with flexural strengths at 7 and 28 days being 28.01% and 29.73% higher than those of the control group, respectively. This suggests that an appropriate combination of fiber length and content can effectively enhance flexural performance. From a mechanistic perspective, the improvement in flexural strength is mainly associated with fiber bridging and energy dissipation during crack propagation. Under bending loading, tensile stresses develop rapidly in the matrix, and properly dispersed fibers can bridge microcracks and delay their coalescence into macrocracks. At the same time, when the fiber dosage is maintained within a suitable range, the matrix continuity and compactness are not significantly disturbed, which allows the reinforcing effect of the fibers to be effectively mobilized.

In contrast, the improvements in A_1_B_1_C_1_D_1_ and A_1_B_3_C_3_D_3_ were less pronounced, with the 28-day flexural strength increasing by only 4.87% and 9.97%, respectively, relative to the control group. For A_1_B_1_C_1_D_1_, the relatively low fiber content limits the number of effective crack-bridging points within the matrix, resulting in an insufficient enhancement of load transfer and crack resistance. For A_1_B_3_C_3_D_3_, although the fiber content is relatively high, excessive fiber incorporation tends to reduce dispersion uniformity and promote agglomeration, which may introduce local weak zones and impair matrix integrity. Therefore, the flexural performance is governed by the balance between fiber reinforcement efficiency and matrix compactness, rather than by fiber dosage alone.

### 3.2. Range Analysis and Ranking of Influencing Factors

The range analysis results for the mechanical properties of RCG are shown in Table 4. As indicated in Table 4, BF length exerts a significantly greater influence on strength than other factors, because BF-reinforced composites enhance crack bridging and stress transfer capabilities [29]. Comparative analysis reveals that BF exerts a greater influence on mechanical properties than PPF at the 7-day age. This is because the RCG gel has not fully developed a continuous, dense three-dimensional network structure at this early stage, resulting in lower strength. At this time, the high-stiffness BF plays the main role in force transmission and constraint, while the elastic modulus of PPF is low. Its reinforcement effect is more reflected in the adjustment of local strain and the constraint of microcracks, and its influence on strength is limited. At the 28-day age, BF length significantly influences strength. Compared with the 7-day curing age, both PPF length and content exert greater effects on strength, particularly in flexural performance, where the effect increased. Because PPF has good ductility and deformation properties, it can absorb more energy in the crack propagation stage by means of destructive deformation and frictional energy dissipation, thereby improving the later strength of the material. Consequently, fiber reinforcement does not remain the same during strength development of the matrix. In the early stage, stiff fibers mainly carry the load, while ductile fibers help restrict crack formation. At later ages, different fibers begin to work together in controlling cracks, which leads to higher strength of the material.

**Figure 6:**
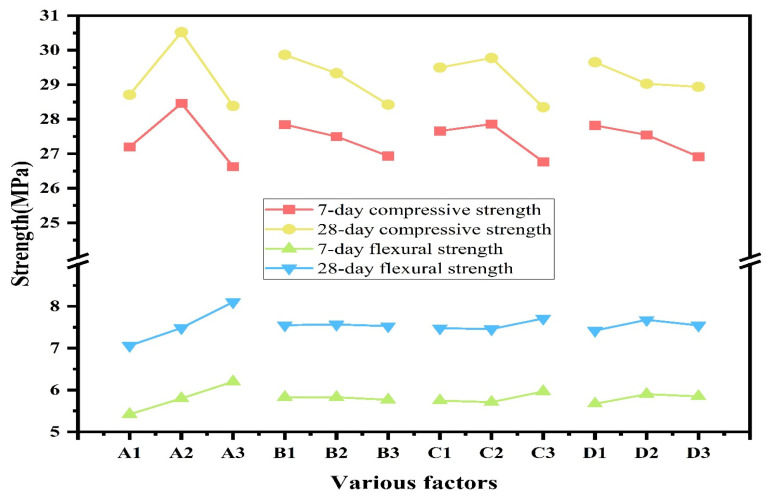
Effects of different factors on compressive and flexural strength.

The effects of various factors on the mechanical properties of fiber-reinforced RCG are shown in Figure 6. As shown in Figure 6, the compressive strength first increases and then decreases with BF length increasing, and the optimal length is 9 mm. At this length, the fibers enhance crack bridging and confinement effects. Meanwhile, the flexural strength increases with increasing BF length. This is because, compared with short fibers, longer BFs possess greater effective anchorage length within the matrix. Longer BFs enhance stress transfer efficiency at the fiber–matrix interface, enabling them to more fully bear the tensile stress across the fracture surface during crack propagation. With an increase in embedment length, fibers are more likely to attain or exceed their critical length, allowing them to reach their ultimate tensile strength [30]. During crack propagation, interfacial failure transitions from simple debonding with short fibers to the stabilized pull-out with long fibers. In this process, the interface friction slip, pull-out resistance, and crack bridging tension work together to increase the energy required for crack propagation. Furthermore, longer fibers can span more cracks, maintaining higher bridging stress levels during crack opening. This mechanism delays the penetration and propagation of the main crack.

PPF has a relatively low elastic modulus and tensile strength compared to BF, which limits the bridging stress. Further, it can still sustain tensile stress under bending. However, its influence on compressive strength is more significant, mainly manifested in the synergistic constraint of cracks when the length is short, so as to improve the material’s strength.

With the increasing of BF content, an optimal content range for compressive strength can be found. With volumetric content changes from 0.1% to 0.2%, the compressive strength increases significantly. After the content exceeds 0.2%, compressive strength shows a downward trend, reflecting that excessive fiber affects the matrix density and the fiber dispersion effect in the matrix, forming local weak areas. Flexural strength also has a peak value, but the position is different from the compressive peak value. The BF specimen with a content of 0.3% has the highest flexural strength. As the volumetric content of PPF increases from 0.1% to 0.3%, the compressive strength decreases, but the decrease at 28 days is significantly less than that at 7 days.

### 3.3. Results of Analysis of Variance

Based on the statistical analysis method described in Section 2.4, an analysis of variance (ANOVA) was performed on the 28-day RCG test data. As listed in Table 5, the significance of the four factors differs for the two response variables. For compressive strength, factor A (BF length) shows the strongest effect (F = 14.031, *p* = 0.001), indicating a highly significant contribution. Factors B (PPF length) and C (BF volume fraction) are also statistically significant (*p* = 0.013 and *p* = 0.011), whereas factor D (PPF volume fraction) is not significant at the 0.05 level (*p* = 0.230). These results indicate that the 28-day compressive strength is mainly governed by the geometric and dosage parameters of basalt fiber, while the influence of polypropylene fiber volume fraction is less pronounced. For flexural strength, factor A remains the dominant variable (F = 24.303, *p* = 0.001), confirming that BF length plays a decisive role in crack bridging and tensile stress transfer under bending. In contrast, factors B, C, and D are not statistically significant at the 95% confidence level (*p* = 0.966, 0.198, and 0.260, respectively). This indicates that, among the investigated factors, the flexural response is most sensitive to BF length, whereas the effects of the other factors are comparatively weaker in statistical terms.

The ANOVA results are consistent with the observed strengthening mechanisms. Basalt fiber has a much higher elastic modulus and tensile strength than polypropylene fiber; therefore, changes in BF length directly affect the effective anchorage length, crack-bridging efficiency, and stress-transfer capability in the matrix. This explains why factor A shows the highest significance for both compressive and flexural strength. By contrast, polypropylene fiber mainly contributes through crack restraint and energy dissipation during deformation. Its reinforcing effect is therefore more closely associated with toughness enhancement and crack-control behavior, rather than showing the same level of direct statistical contribution to strength as BF length.

Overall, the ANOVA results are broadly consistent with the range analysis, especially in confirming the dominant role of BF length. In addition, the coefficients of determination of the ANOVA models are R^2^ = 0.752 for compressive strength and R^2^ = 0.764 for flexural strength, indicating that the selected factors account for a considerable proportion of the variation in the responses. Moreover, the statistical analysis further supports the conclusion that optimizing BF length is the key to improving the mechanical performance of hybrid fiber-reinforced RCG, while the effects of the remaining factors depend on the specific mechanical index considered.

### 3.4. Strength Response Surface Model and Optimal Fiber Parameters

**Figure 7:**
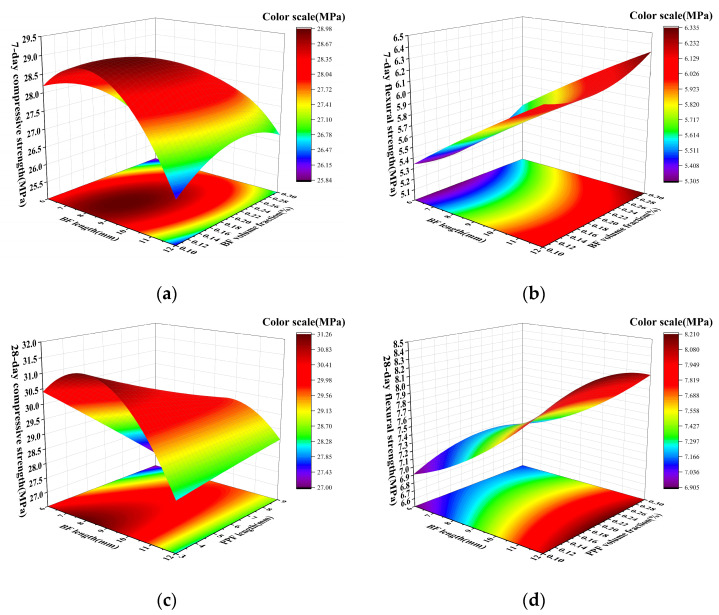
Response surface plots of strength versus significant factors: (**a**) 7-day compressive strength; (**b**) 7-day flexural strength; (**c**) 28-day compressive strength; (**d**) 28-day flexural strength.

Response surface methodology (RSM) has noteworthy advantages in gaining a deeper understanding of the mechanisms of multi-factor interactions and in designing optimal combinations. It can be combined with analysis of variance (ANOVA) to test the significance of the model and factors [30] and it has been widely applied in studies on mix design optimization and mechanical property prediction for geopolymers and similar cementitious systems [31,32,33]. As shown in Figure 7, based on experimental data, the two factors with the highest influence rankings from different experiments in the range analysis section were selected to plot response surface diagrams for strength response surface plots.

As shown in Figure 7,

(1) The 7-day compressive strength shows a trend of first increasing and then decreasing with the increase of BF length and volumetric content. Compared with BF lengths of 6 mm and 12 mm, the compressive strength of 9 mm BF increases by 5% and 9%, respectively. During the increase of volumetric content, the response surface becomes gentler, reaching a peak near 0.14% and then decreasing, showing a certain threshold effect. The flexural strength shows a significant upward trend with the increase of BF length and volumetric content, and the increase is stronger than that of the compressive strength. It proves that in the early stage, the fiber parameters are significantly more sensitive to the flexural performance than to the compressive performance. The reason is that flexural failure belongs to the tensile-controlled failure mode and the microcracks inside the material expand rapidly under the action of bending tensile stress, while BF with greater stiffness can share more tensile stress near the internal cracks, thereby improving the flexural strength.
(2) By comparing the 7-day response curves of compressive and flexural strength in Figure 7a,b, it is found that the length of BF should be controlled within 9 to 12 mm and the volumetric content should be maintained within 0.10% to 0.24% in order to balance the trade-off between the density of the matrix and the fiber bridging performance. At this time, the best comprehensive mechanical properties can be obtained.
(3) As shown in Figure 7c, the compressive strength of 28 d is higher than that of 7 d, and its strength exhibits a “first increase, then decrease” trend with the change of BF length, but the peak region of the response surface is flatter. This proves that after the matrix structure stabilizes, the influence of fiber parameters on compressive performance weakens. Blending fibers within a reasonable range can achieve good compressive performance, but when the length exceeds 10 mm, the increased fiber agglomeration and interfacial transition zone (ITZ) may weaken the matrix continuity, resulting in a slight decrease in strength.
(4) As shown in Figure 7d, the flexural strength at 28 d increases monotonically with the increase of fiber length and volume fraction. When the strength increases from 6.9 MPa to 8.2 MPa, the increase is close to 20%. Additionally, fiber length has a stronger impact on 28-day flexural strength compared with the volume fraction of PPF. In particular, the strength increase is more significant when the BF length increases from 6 mm to 12 mm, while the strength change is relatively gradual when the volume fraction increases. Furthermore, the response surface in the higher volumetric content region is flat, but it does not decline overall. Compared with compressive strength, the fiber parameters respond more significantly to flexural strength, indicating that the flexural failure mechanism of RCG remains unchanged with age increasing and is still dominated by tensile failure.

To evaluate the reliability of the response surface models, statistical parameters were analyzed, as summarized in Table 6. The standard deviations of the four responses are relatively small, and the corresponding coefficients of variation (CV) are 1.08%, 2.05%, 1.08%, and 1.79%, respectively, all below 5%. This indicates that the response data show low dispersion and good overall stability. In addition, the R^2^ values of the four models are 0.9563, 0.9121, 0.9611, and 0.9319, respectively, all exceeding 0.90, which demonstrates that the models can adequately describe the relationships between the selected factors and the corresponding responses. Furthermore, the Adjusted R^2^ values are close to the corresponding R^2^ values, indicating that the fitted models maintain good explanatory ability after accounting for the number of terms included. However, a relatively large gap between Adjusted R^2^ and Predicted R^2^ suggests that the predictive performance of the models is less robust than their fitting performance. This result indicates that, although the developed models are effective in capturing the main response trends and factor-response relationships within the investigated parameter domain, caution is still needed when using them for direct prediction of response values, especially for parameter combinations near the boundary of the design space.

Therefore, the present response surface models are more suitable for identifying the relative importance of factors, analyzing response trends, and determining optimal parameter regions, rather than for highly precise point prediction. From this perspective, the models remain effective for interpreting the effects of fiber parameters on the mechanical properties of RCG and for supporting the optimization analysis conducted in this study. In addition, the Adequate Precision values of all models are much greater than 4, indicating an adequate signal-to-noise ratio and confirming that the models are acceptable for response surface analysis and process optimization.

To find the optimal mix proportion for geopolymers, compressive strength and flexural strength were jointly used as the primary optimization objectives for mechanical properties. Based on response surface analysis results, Design-Expert software (version 13.0.1.0) predicted the optimal factor level combinations for these two curing ages: (1) 7-day age: BF length 10 mm, volume fraction 0.22%; PPF length 3 mm, volume fraction 0.15%. (2) 28-day age: BF length 11 mm, volume fraction 0.23%; PPF length 6 mm, volume fraction 0.2%.

### 3.5. Fiber Reinforcement Mechanism

#### 3.5.1. SEM-EDS

**Figure 8:**
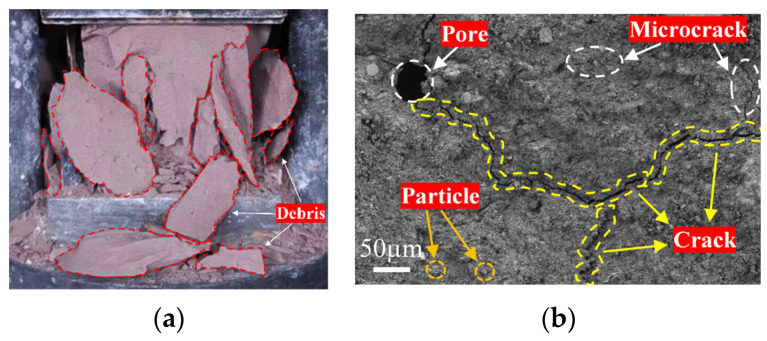
Failure characteristics of plain RCG: (**a**) macroscopic brittle failure; (**b**) microstructure after failure.

The macroscopic fracture morphology of unreinforced geopolymer is shown in Figure 8a, which exhibits typical brittle failure. After being subjected to compression, the specimen rapidly fractured, forming a through-crack. The fracture surface was smooth, with regular debris. From the microstructure (Figure 8b), numerous pores and incompletely reacted particles exist within the matrix, which have formed a network of interconnected microcracks. Most of these cracks originate at pore interfaces and propagate to form interconnected networks, compromising material integrity and readily inducing stress concentrations ultimately trigger macroscopic brittle fracture.

**Figure 9:**
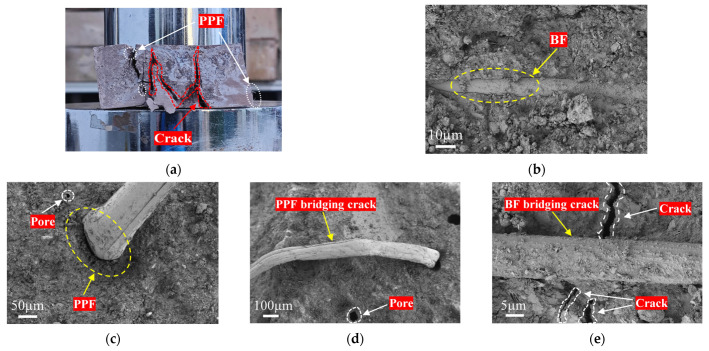
Failure characteristics of hybrid fiber-reinforced RCG: (**a**) macroscopic ductile failure; (**b**) BF bonding; (**c**) PPF bonding; (**d**) PPF crack-bridging; (**e**) BF crack-bridging.

Figure 9 shows the failure morphology of fiber-reinforced RCG. It proves that the failure mode changes from brittle to ductile after fiber addition. As shown in Figure 9a, the specimen remained relatively intact after failure, exhibiting a rough fracture surface. Microscopic images (Figure 9b,c) show that both basalt fibers (BFs) and polypropylene fibers (PPFs) form a strong interfacial bond with the RCG matrix, without significant debonding or through-pores, indicating that both fibers effectively participate in stress transfer within the matrix. Meanwhile, this study also observes significant fiber bridging along the crack propagation paths (Figure 9d,e).

**Figure 10:**
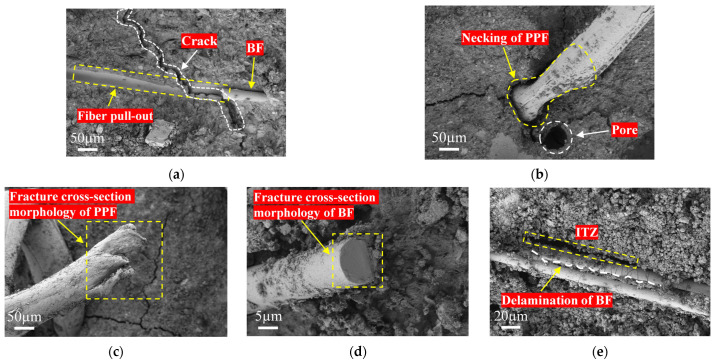
Failure modes of fibers: (**a**) BF pull-out failure; (**b**) PPF necking failure; (**c**) fracture surface of PPF; (**d**) Fracture surface of BF; (**e**) BF delamination and ITZ.

Figure 10 shows typical failure modes of fibers in RCG. The fiber-bridging mechanism varies depending on the fiber type. Figure 10a,b show that the failure modes of PPF and BF differ significantly: PPF mainly fractures after plastic deformation, demonstrating ductile failure characteristics; BF typically demonstrates brittle fracture with fiber pull-out or interfacial debonding. The interfacial bonding condition is a key for regulating failure modes: when bonding is fragile, fibers are prone to being pulled out, confirmed by interface slip traces in Figure 10a. Even with strong bonding, crack propagation may trigger controlled fiber pull-out, a process that dissipates energy through friction. When the interfacial bond strength is sufficient, stress will be directly transmitted to the fiber body, leading to fracture. As reflected in Figure 10c,d, the PPF fracture surface exhibits a coarse torn morphology and pronounced necking, indicating energy dissipation through plastic deformation. In contrast, BF exhibits a smooth fracture surface characteristic of typical brittle fracture (Figure 10e).

In summary, BF primarily relies on its high modulus to provide rigid bridging constraints during the early crack stage, with failure modes mainly involving fiber pull-out or brittle fracture. In contrast, PPF dissipates energy through plastic deformation followed by tensile fracture. The synergistic effect of the two fibers transforms crack propagation from a single rapid penetration process into a multi-stage, high-energy-absorption process through the combination of rigid bridging and plastic energy dissipation. As a result, the crack resistance and overall mechanical strength of RCG composite materials are significantly enhanced.

**Figure 11:**
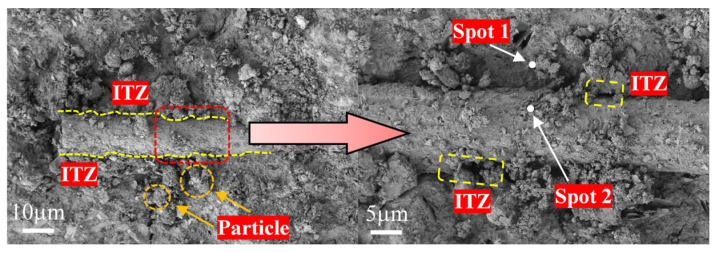
ITZ and selected EDS test points.

**Figure 12:**
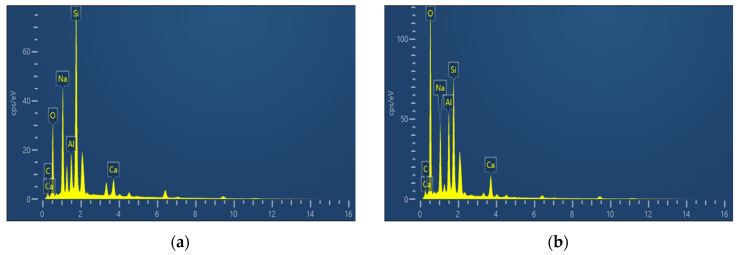
EDS spectra at selected points: (**a**) Spot 1; (**b**) Spot 2.

Under SEM observation, an interfacial transition zone (ITZ) can be identified between the RCG matrix and the fibers, as shown in Figure 10e and Figure 11. To further examine the interfacial characteristics, EDS point analysis and line-scan analysis were conducted to evaluate the elemental distribution and variation across the interface. The purpose of this analysis is not to determine the absolute composition of the interface, but to characterize the relative elemental transition across the ITZ. Two representative locations, namely the fiber region (Spot1) and the matrix region (Spot2), were selected for comparison, and the corresponding spectra are presented in Figure 12.

EDS point analysis suggests clear differences in elemental characteristics between the two sampling points. In the fiber region (Spot1), the Si signal is relatively strong, while signals of elements such as Al, Ca, and Fe associated with the geopolymer matrix are also detected. This is consistent with the chemical composition characteristics of the fiber and suggests possible elemental interaction between the matrix and the fiber. In the matrix region (Spot2), the peak intensity of oxygen is significant, while signals from matrix elements such as Si, Al, and Na are clearly discernible. Combined with XRD results, this suggests the formation of aluminosilicate gel under alkaline activation. Compared with Spot1, the Si peak height at Spot2 is slightly lower, showing a gradual transition in Si distribution from BF fibers to the matrix. The presence of Na further supports the identification of the matrix region. The aforementioned differences indicate the material composition on either side of the ITZ at the microscopic chemical level: one side corresponds to the Si-rich fiber, while the other comprises a geopolymer matrix rich in O and Na. Elements of the matrix were also detected on the fiber surface, suggesting interaction at the interface. In combination with the SEM observations, no obvious pores or microcracks are observed at the interface, and the fiber surface appears to be well bonded to the surrounding matrix gel, indicating a relatively dense and continuous ITZ structure. Such an interfacial structure is beneficial for stress transfer between the fibers and the matrix, providing a crucial microscopic foundation for the enhancement of macroscopic mechanical properties.

Therefore, the role of the ITZ in this system should be understood as a coupling zone between the matrix and the fibers rather than a simple geometric boundary. A relatively dense and continuous ITZ can help delay interfacial debonding and improve load transfer efficiency, thereby contributing to the enhancement of macroscopic mechanical properties. However, this interpretation is based on combined SEM, EDS, and mechanical observations, and EDS is used here only as a semi-quantitative tool for elemental distribution analysis [34].

**Figure 13:**
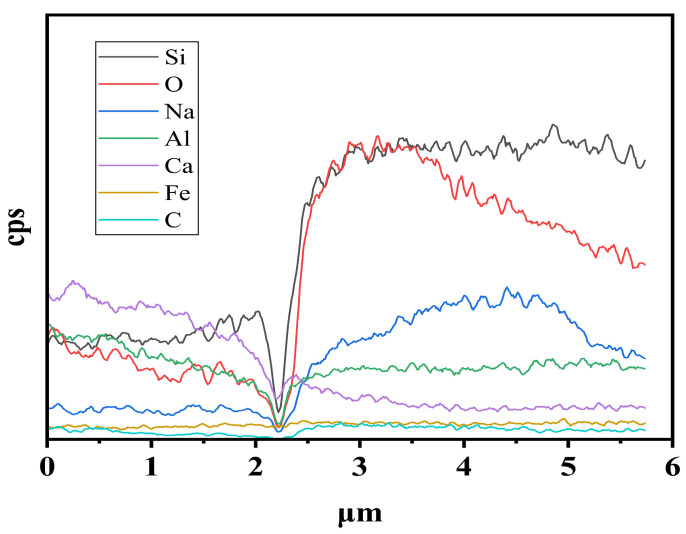
EDS line-scan results across the fiber–matrix interface.

Within a 5 μm range at the fiber–matrix interface, line scan analysis was conducted. The results in Figure 13 suggest the presence of a distinct interfacial transition zone within the material. As the scan position approaches the matrix, the Fe signal gradually weakens, while the signals from characteristic elements of the matrix phase, such as Si, Al, and Ca, gradually increase. A minor C signal is also detected, which is attributed to the use of carbon-based modes during the sample preparation process, rather than being part of the geopolymer matrix. The Si and Al elements exhibit a continuous and smooth transition at the interface, and there are no abrupt signal drop or element-depleted zones observed. This suggests that the fiber and matrix have formed a relatively tight mechanical interlocking, and the ITZ structure is stable. Simultaneously, Ca enrichment is observed near the interface, which promotes the preferential formation of gel structures in the interfacial region and enhances chemical compatibility. The surface of basalt fibers is rich in Si, exhibiting excellent affinity with the geopolymer matrix. This may facilitate the formation of a stable bonding layer, enabling effective stress transfer across the interface.

**Figure 14:**
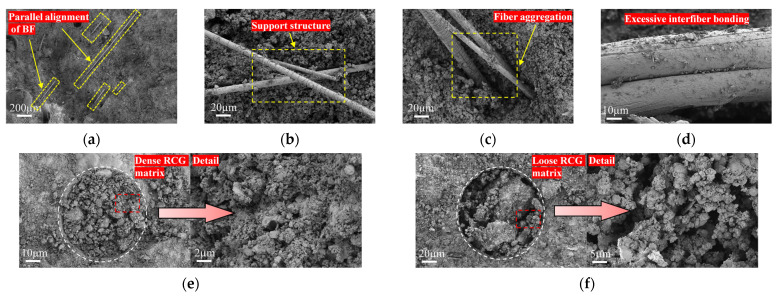
Comparison of microstructural morphology: (**a**) uniform fiber distribution; (**b**) support structure; (**c**) fiber aggregation; (**d**) poor fiber dispersion; (**e**) dense RCG matrix; (**f**) loose RCG matrix.

Furthermore, SEM analysis was conducted to further verify with the group of minimum and maximum micromechanical strengths: A_3_B_1_C_3_D_2_ and A_1_B_3_C_3_D_3_. As shown in Figure 14, these two sets exhibit distinct differences in fiber distribution patterns and matrix structural density.

In A_3_B_1_C_3_D_2_ (Figure 14a,b,e), SEM observations show that BF is relatively uniformly distributed and tends to be aligned in a roughly parallel manner within the matrix. Such a fiber arrangement is favorable for forming effective load-transfer paths in multiple directions. In addition, some local regions exhibit a relatively dense RCG matrix, in which gel products are more continuously distributed and fewer pores are observed. The gel particles appear closely packed, suggesting a relatively higher degree of geopolymerization. These microstructural features, namely a more uniform fiber distribution together with a denser matrix structure, are consistent with the superior mechanical performance of this group. In particular, compared with the control group, the 28-day flexural strength of A_3_B_1_C_3_D_2_ increased by 29.73%, indicating that the reinforcing effect of the fibers was effectively mobilized under this parameter combination.

In contrast, A_1_B_3_C_3_D_3_ (Figure 14c,d,f) exhibits obvious fiber agglomeration and excessive fiber-to-fiber contact. In localized areas, inefficient fiber stacking and mutual adhesion are observed, which makes it difficult for the matrix to fully fill the voids between fibers and thus introduces structural defects. In addition, Figure 14f shows that the RCG matrix in this group is relatively loose, with more pores and uneven gel coverage. These observations suggest that excessive fiber content interferes with matrix densification and reduces structural continuity. Fiber agglomeration not only lowers the effective utilization of fibers, but may also create localized stress concentration regions, making crack initiation and propagation more likely. This interpretation is consistent with the mechanical results: compared with the control group, the 28-day flexural strength of A_1_B_3_C_3_D_3_ increased only by 9.97%, while its 7-day and 28-day compressive strengths decreased by 2.25% and 3.34%, respectively. Therefore, although fiber addition is generally beneficial for reinforcement, excessive fiber dosage can deteriorate both fiber dispersion and matrix compactness, ultimately offsetting the positive reinforcing effect.

Although no image-based dispersion coefficient was determined in the present study, the comparative SEM observations combined with the corresponding strength differences provide semi-quantitative evidence that fiber dispersion and matrix compactness jointly govern the reinforcing efficiency.

Overall, SEM analysis indicates that the fiber reinforcement efficiency is governed not only by the fiber dispersion state, but also by the quality of the fiber–matrix interface. Within a reasonable range of fiber content, no obvious pores or fracture zones are observed inside the matrix. The crack propagation paths are restricted, and the material continuity remains stable. With a further increase in the fiber content, the interface integrity in localized regions deteriorates. Cracks then propagate preferentially along weak or defective paths, leading to a reduction in reinforcement efficiency. Further, there is an effective range for coordination between fiber content and matrix structure. Its essence is the balance between the integrity of the interface structure and the fiber bridging efficiency, rather than being simply determined by the dispersion state.

#### 3.5.2. XRD Result

**Figure 15:**
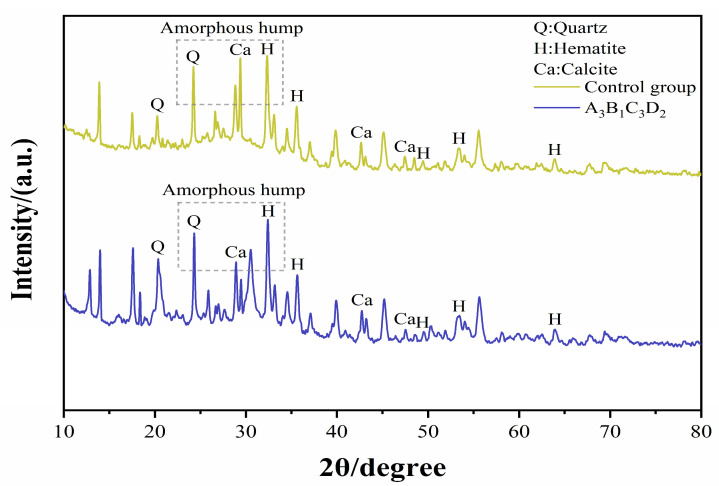
XRD patterns of RCG and hybrid fiber-reinforced RCG.

XRD analysis was conducted on the Control group and A_3_B_1_C_3_D_2_ specimens at 28 days. The results are shown in Figure 15. The XRD patterns exhibit a high degree of similarity between the plain and fiber-reinforced samples. This demonstrates that the fundamental phase assemblage of the system is not altered by the fibers. The mineral phases include quartz, hematite, and calcite. It originates from residual mineral components in RM and CMK. These phases are inert during geopolymerization. Furthermore, quartz peaks were observed in both sets of samples, exhibiting relatively high intensities in the 20–30° (2θ) range, consistent with the findings reported by Uysal [35], Nazir [16].

In addition, a broad hump is observed within the range of 20–35°. This region corresponds to the characteristic peak of an amorphous aluminosilicate gel structure. This confirms the formation of substantial amounts of C-A-S-H or N-A-S-H gel products within the geopolymer matrix, with the EDS observation of Ca element enrichment near the interface.

**Figure 16:**
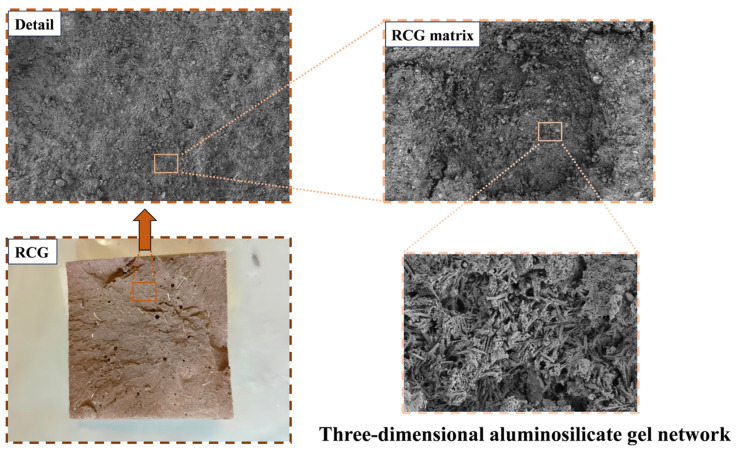
Three-dimensional network structure of aluminosilicate gel.

SEM images of A_3_B_1_C_3_D_2_ reveal the formation of extensive, interwoven aluminosilicate gel networks. These gels are interconnected, constituting a continuous three-dimensional skeletal structure, as illustrated in Figure 16. The structure effectively dampens pore formation, reinforcing the density and integrity of the matrix structure. It provides an explanation for the mechanical strength and demonstrates the designed mix proportion can achieve effective geopolymerization. Meanwhile, compared with the control group, the dispersion peak of the fiber-reinforced specimens shifts slightly, indicating that incorporating fibers within an appropriate range can enhance matrix densification and promote a more uniform gel distribution.

## 4. Discussion

This study systematically analyzed the mechanical properties and mechanisms of BF and PPF hybrid reinforcement in RCG. The results showed that hybrid fibers significantly improved the compressive strength and flexural strength of RCG, with BF length being the dominant factor affecting the mechanical properties. The BF content and PPF length had secondary effects, while the PPF content had a relatively minor influence. Response surface analysis further identified the optimal fiber combination parameters, and microstructural analysis confirmed the fiber bridging, interface stress transfer, and crack inhibition mechanisms. Overall, hybrid fibers not only increased the strength of RCG but also improved its crack resistance and structural stability, indicating that this reinforcement strategy has high engineering application potential in the optimization of industrial waste-based geopolymer materials.

Previous studies have also confirmed that BF can effectively enhance the mechanical properties and crack control capabilities of geopolymer materials. For example, one study indicated that the incorporation of different amounts of BF in fly ash-based geopolymer led to an increase in compressive strength by approximately 6.43–11.94% and an increase in flexural strength by approximately 7.72–34.15%, suggesting that basalt fiber has a more significant effect on improving flexural performance [36]. Another study found that the inclusion of different amounts of basalt fiber in a silicate-based geopolymer system resulted in an increase in strength by about 10–34%, demonstrating that the fiber bridging effect can effectively enhance the material’s load-bearing capacity [37]. These results are consistent with the conclusion of this study, where fibers significantly improved the mechanical properties of RCG. In hybrid fiber systems, previous studies have pointed out that there is a significant synergistic enhancement effect between BF and PPF, which can improve the material’s physical and mechanical properties and enhance structural stability [38]. Furthermore, related research has shown that the introduction of basalt fiber can significantly improve the compressive toughness and energy absorption capacity of geopolymer materials. Under appropriate fiber content and length conditions, the compressive toughness can increase by more than 100%, indicating that the fiber bridging and crack inhibition effects can effectively improve the material’s failure mode [39]. Therefore, the results of the synergistic enhancement of RCG’s mechanical properties by BF and PPF in this study are in good agreement with previous research. From the perspective of enhancement mechanisms, current studies generally agree that basalt fibers can enhance the performance of geopolymer materials through crack bridging, interface stress transfer, and increased fracture toughness. For example, a study using fracture mechanics analysis and SEM observation found that BF significantly improve the fracture toughness and crack propagation resistance of geopolymer, forming a stable fiber bridging structure during the failure process [40]. Furthermore, related studies have pointed out that the interface bonding and physical interlocking between the fibers and the geopolymer matrix are the primary reasons for the enhanced strength and crack resistance of the material [41]. Additionally, research has shown that the fiber reinforcement effect is closely related to the density of the matrix structure, and there exists an optimal fiber content range [42]. These findings are consistent with the significant trends of fiber reinforcement revealed in the ANOVA and response surface analysis in this study.

Our experimental results show that the hybrid incorporation of BF and PPF significantly improves the mechanical properties of RCG, especially flexural strength and crack resistance. This enhancement is mainly attributed to the synergistic effect of the two fibers. Basalt fiber has high tensile strength and elastic modulus, enabling it to restrict macrocrack propagation and enhance compressive performance of geopolymer composites [39,40]. In contrast, polypropylene fiber mainly contributes to microcrack control and toughness improvement through pull-out and energy dissipation mechanisms, which has been widely reported in hybrid fiber geopolymer systems [17,18,27,28].

The combination of BF and PPF provides a complementary reinforcement mechanism, in which BF controls macrocrack development while PPF restrains microcrack initiation, resulting in multi-scale crack control and improved mechanical performance. Similar synergistic effects have been observed in hybrid fiber cementitious composites [29,42]. SEM observations in this study further confirm this mechanism, showing basalt fiber bridging and polypropylene fiber pull-out behavior, which enhance interfacial bonding and energy dissipation during fracture. These results are consistent with the existing literature.

Overall, the results of this study are in good agreement with existing literature in terms of the improvement in mechanical properties and enhancement mechanisms. At the same time, the hybrid fiber reinforcement method has been extended to the red mud–coal metakaolin geopolymer system, further validating the applicability of fiber reinforcement technology in complex industrial waste-based materials. This study also provides new experimental evidence for optimizing the fiber parameters in red mud-based geopolymer composites. However, this study has certain limitations. First and foremost, the experiments primarily focused on the mechanical performance analysis under room temperature curing conditions, without systematically investigating the performance changes under long-term durability or environmental exposure. Furthermore, the response surface model was developed based on a limited range of parameters, and its applicability still needs to be validated through experiments over a wider range of conditions. Future research could further investigate the durability of hybrid fibers under high-temperature, freeze–thaw, and erosive environments. Additionally, combining multi-scale simulations and machine learning methods to optimize fiber parameter design could help advance the practical application of red mud-based geopolymer materials in engineering structures.

## 5. Conclusions

The hybrid incorporation of basalt fiber (BF) and polypropylene fiber (PPF) effectively improved the mechanical behavior of red mud–coal metakaolin geopolymer (RCG), but the enhancement was response-dependent. The gain in flexural strength was more pronounced than that in compressive strength, indicating that the primary contribution of hybrid fibers lies in crack control, failure-mode regulation, and toughness enhancement, rather than in a simple increase in load-bearing capacity.

Among the investigated factors, BF length was identified as the governing design parameter for both compressive and flexural performance, while BF dosage showed a distinct optimum range. In contrast, PPF contributed less to early-age strength, whereas Its influence on crack control and toughness enhancement has become more apparent with increasing curing age.

The optimal fiber combination (11 mm BF length, 0.23% BF volume fraction; 6 mm PPF length, 0.2% PPF volume fraction) obtained from the response surface analysis reflects a balance between fiber reinforcement efficiency and matrix integrity. This suggests that the mechanical performance of RCG is not governed by fiber addition alone, but by the coupling of appropriate fiber length, dosage, and dispersion state.

Microstructural analyses revealed that the performance enhancement originated primarily from microstructural regulation instead of phase alteration. XRD showed that fiber incorporation did not fundamentally change the phase assemblage of the geopolymer matrix, whereas SEM-EDS confirmed a relatively dense interfacial transition zone, effective stress transfer, and evident fiber bridging or pull-out behavior. BF mainly acted as a rigid crack-bridging skeleton, while PPF restrained microcrack initiation and dissipated fracture energy through deformation, together transforming rapid brittle cracking into a staged crack-arrest process.

From a design perspective, the present study shows that hybrid fiber reinforcement is a feasible strategy for improving the mechanical reliability of high red-mud geopolymer systems, but the optimization results should be interpreted within the investigated parameter domain. Although the response surface models exhibited good fitting ability, their predictive robustness was lower than their fitting performance. Therefore, they are more suitable for identifying effective parameter regions and revealing factor interactions than for highly precise extrapolative prediction.

## Figures and Tables

**Table 1:** Chemical composition of RM and CMK.

Chemical Composition	SiO_2_	Al_2_O_3_	Fe_2_O_3_	TiO_2_	CaO	MgO	K_2_O	Na_2_O	Loss on Ignition
RM	21.05	27.38	6.42	4.04	14.91	0.53	0.77	11.86	8.78
CMK	54.23	41.11	1.28	0.63	0.12	0.12	1.31	0.23	—

**Table 2:** Physical and mechanical properties of fibers (provided by manufacturers).

Type	Diameter/μm	Density/g·cm^3^	Elongation at Break/%	Elastic Modulus/GPa	Tensile Strength/MPa
BF	15	2.64	30	107	2100
PPF	42	0.92	21	4.5	590

**Table 3:** Orthogonal experimental design.

Divisor	BF Length/mm	PPF Length/mm	BF Volume Content/%	PPF Volume Content/%
Test Group				
A_1_B_1_C_1_D_1_	6	3	0.1	0.1
A_1_B_2_C_2_D_2_	6	6	0.2	0.2
A_1_B_3_C_3_D_3_	6	9	0.3	0.3
A_2_B_1_C_2_D_3_	9	3	0.2	0.3
A_2_B_2_C_3_D_1_	9	6	0.3	0.1
A_2_B_3_C_1_D_2_	9	9	0.1	0.2
A_3_B_1_C_3_D_2_	12	3	0.3	0.2
A_3_B_2_C_1_D_3_	12	6	0.1	0.3
A_3_B_3_C_2_D_1_	12	9	0.2	0.1
Control	—	—	—	—

**Table 4:** Range analysis of mechanical properties of RCG with hybrid fibers.

Index	Factor	A	B	C	D	Significance Order of Factors	Index	Factor	A	B	C	D	Significance Order of Factors
7-day Compressive Strength	R	1.83	0.91	1.09	0.90	A > C > B > D	7-day Flexural Strength	R	0.78	0.06	0.26	0.23	A > C > D > B
	Optimal Combination	A_2_	B_1_	C_2_	D_1_			Optimal Combination	A_3_	B_1_	C_3_	D_2_	
28-day Compressive Strength	R	2.14	1.44	1.42	0.71	A > B > C > D	28-day Flexural Strength	R	1.04	0.04	0.25	0.26	A > D > C > B
	Optimal Combination	A_2_	B_1_	C_2_	D_1_			Optimal Combination	A_3_	B_2_	C_3_	D_2_	

**Table 5:** ANOVA for 28-day compressive and flexural strength.

Compressive Strength Factors	Sum of Squares	Mean Square	*df*	F Value	*p* Value	Flexural Strength Factors	Sum of Squares	Mean Square	*df*	F Value	*p* Value
A B C D *R*^2^ = 0.752	23.879	11.939	2	14.031	0.001	A B C D *R*^2^ = 0.764	4.943	2.472	2	24.303	0.001
	9.522	4.761	2	5.595	0.013		0.007	0.003	2	0.034	0.966
	10.285	5.143	2	6.043	0.011		0.360	0.180	2	1.772	0.198
	2.713	1.356	2	1.594	0.230		0.296	0.148	2	1.454	0.260

**Table 6:** Statistical parameters of the developed response surface models.

Parameter	7 d Compressive	7 d Flexural	28 d Compressive	28 d Flexural
Standard Deviation	0.2991	0.1189	0.3200	0.1349
Mean	27.81	5.80	29.53	7.54
CV (%)	1.08	2.05	1.08	1.79
R^2^	0.9563	0.9121	0.9611	0.9319
Adjusted R^2^	0.9251	0.8494	0.9333	0.8833
Predicted R^2^	0.5827	0.2272	0.7119	0.3609
Adequate Precision	14.7230	12.3719	18.6805	14.1638

## Data Availability

The original contributions presented in this study are included in the article. Further inquiries can be directed to the corresponding author.
